# ﻿Four new species of leptonetid spiders (Araneae, Leptonetidae) from Anhui Province, China

**DOI:** 10.3897/zookeys.1218.136555

**Published:** 2024-11-18

**Authors:** Shuhui Li, Qiang Chen, Yanfeng Tong

**Affiliations:** 1 College of Life Science, Shenyang Normal University, Shenyang 110034, China Shenyang Normal University Shenyang China; 2 Experimental Teaching Center, Shenyang Normal University, Shenyang 110034, China Shenyang Normal University Shenyang China

**Keywords:** Asia, biodiversity, identification key, *Jingneta*, *Leptonetela*, new taxa, *Rhyssoleptoneta*, taxonomy

## Abstract

Four new species of leptonetid spiders from Anhui Province, China are recognized: *Jingnetaqishan* Tong, **sp. nov.** (♂♀), *Jingnetawukuishan* Tong, **sp. nov.** (♂), *Leptonetelajingde* Tong, **sp. nov.** (♂♀) and *Rhyssoleptonetalishan* Tong, **sp. nov.** (♂♀). An identification key to leptonetid spiders from Anhui is provided.

## ﻿Introduction

Members of the family Leptonetidae Simon, 1890 are tiny (1–3 mm) and typically have six eyes, with the posterior median eyes displaced behind the anterior lateral eyes and the posterior lateral eyes, and anterior median eyes have been lost. Most species live in moist habitats, such as leaf litter, under rocks and in caves (Ledford et al. 2021).

Leptonetidae includes 22 genera and 392 species from North America, the Mediterranean, and East and Southeast Asia (World Spider Catalog 2024). Currently, 145 species belonging to eight genera have been recorded in China (Wang et al. 2020; Liu and Zhang 2022; Yang et al. 2022; Hu and Liu 2023; Liu et al. 2024). Anhui Province, a provincial-level administrative region of China, is located in the Yangtze River Delta region of East China (Fig. 14). Four species belonging to three genera have been recorded in Anhui Province: *Jingnetamaculosa* (Song & Xu, 1986), *J.tunxiensis* (Song & Xu, 1986), *Leptonetelamicrodonta* (Xu & Song, 1983) and *Longileptonetashenxian* Wang & Li, 2020 (Xu and Song 1983; Song and Xu 1986; Wang et al. 2020).

In this study, four new species of leptonetid spiders from the Anhui Province of China are described and illustrated. An identification key is provided.

## ﻿Materials and methods

Specimens used in this study were collected by sifting forest leaf litter and examined using a Leica M205 C stereomicroscope. Fine details were studied using an Olympus BX51 compound microscope. Female genitalia were cleared in lactic acid. Photomicroscope images were made with a Canon EOS 750D zoom digital camera (24.2 megapixels) mounted on an Olympus BX51 compound microscope. Photos were stacked with Helicon Focus ® (version 8.2.0) and processed in Adobe Photoshop CC 2020 ®. Scanning electron microscope images (SEM) were taken under high vacuum with a Hitachi S-4800 after critical-point drying and gold-palladium coating. Leg measurements are shown as: total length (femur, patella, tibia, metatarsus, tarsus) and, when missing, were coded as “–”. All measurements were taken using an Olympus BX51 compound microscope and are in millimeters.

All specimens are preserved in 75% ethanol. The type material is deposited in the Shenyang Normal University (**SYNU**) in Liaoning, China (curator: Yanfeng Tong).

Terminology follows Wang et al. (2020) and Yang et al. (2022). The following abbreviations are used in the text and figures: AER = anterior eye row; ALE = anterior lateral eyes; at = atrium; emb = embolus; ma = median apophysis; mo = median outgrowth; ms = median sclerite; PER = posterior eye row; pl = prolateral lobe; PLE = posterior lateral eyes; PME = posterior median eyes; po = prolateral outgrowth; ps = prolateral sclerite; rl = retrolateral lobe; ro = retrolateral outgrowth; sc = scape; so = small outgrowth; sp = spermathecae; spr = short projection; ss = spermathecal stalk; ts = tarsal spur.

## ﻿Taxonomy

### ﻿Family Leptonetidae Simon, 1890

#### ﻿Genus Jingneta

Taxon classificationAnimaliaAraneaeLeptonetidae

Wang & Li, 2020

C2F38099-06F2-5573-B490-107B303B2F88

##### Type species.

*Leptonetacornea* Tong & Li, 2008.

##### Diagnosis.

See Wang et al. (2020).

##### Composition.

Twelve species, including two described here.

##### Distribution.

China (Anhui, Beijing, Hebei).

#### ﻿ Jingneta qishan

Taxon classificationAnimaliaAraneaeLeptonetidae

Tong sp. nov.

BA9BEDA2-52B7-5728-9A91-8E8CDCAF13DB

https://zoobank.org/C77BF2C5-2968-4EAA-9F12-11D412A835DF

**Figure 1.**
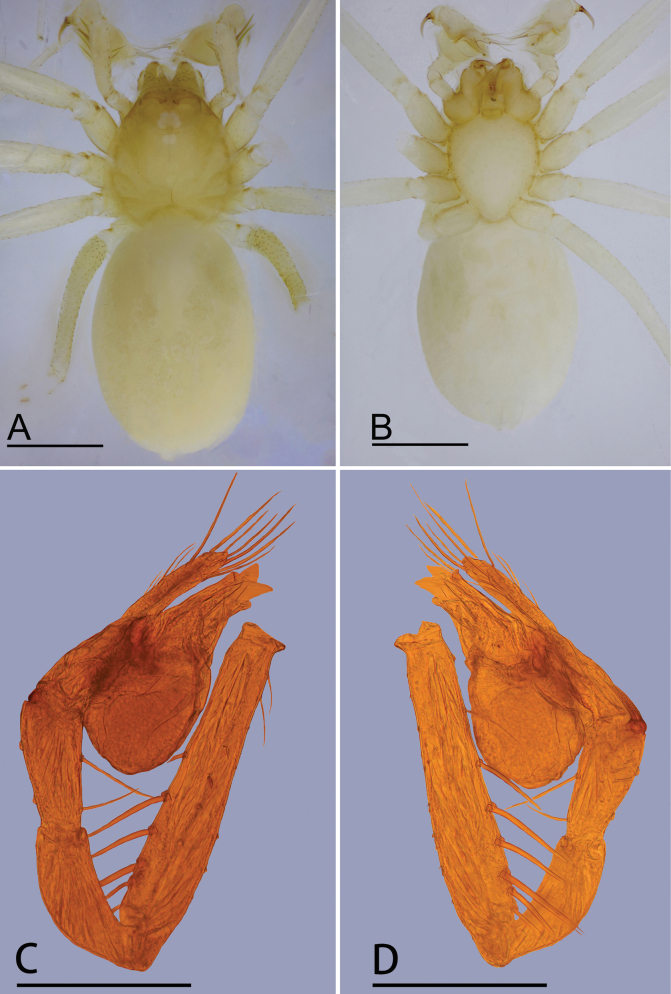
*Jingnetaqishan* sp. nov., male **A, B** habitus, dorsal and ventral views **C, D** left palp, prolateral and retrolateral views. Scale bars: 0.4 mm (**A, B**); 0.3 mm (**C, D**).

**Figure 2.**
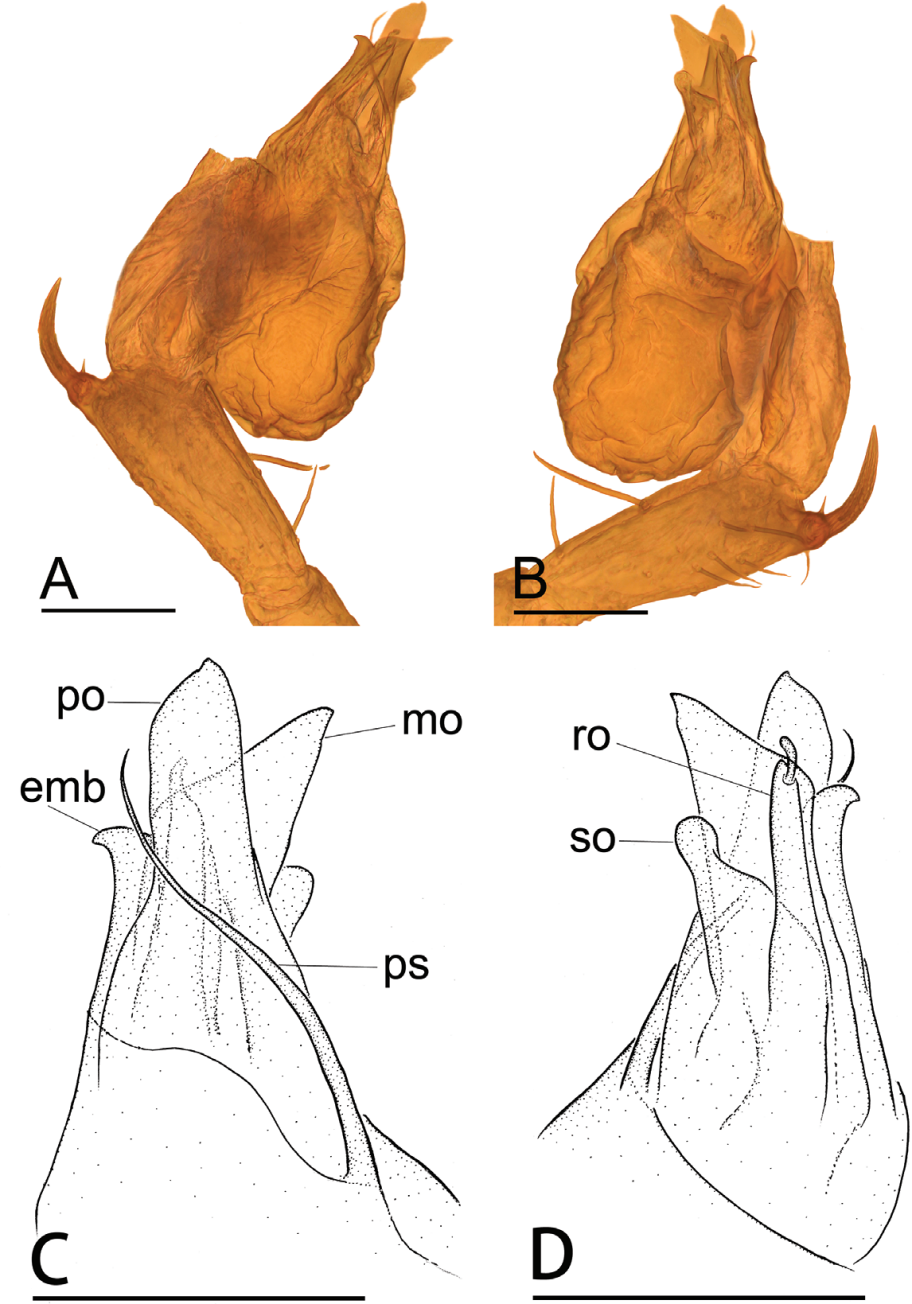
*Jingnetaqishan* sp. nov. **A, B** left palpal bulb and tibia, prolateral and retrolateral views **C, D** distal part of bulb, prolateral and retrolateral views. Abbreviations: emb = embolus; mo = median outgrowth; po = prolateral outgrowth; ps = prolateral sclerite; ro = retrolateral outgrowth; so = small outgrowth. Scale bars: 0.1 mm.

**Figure 3.**
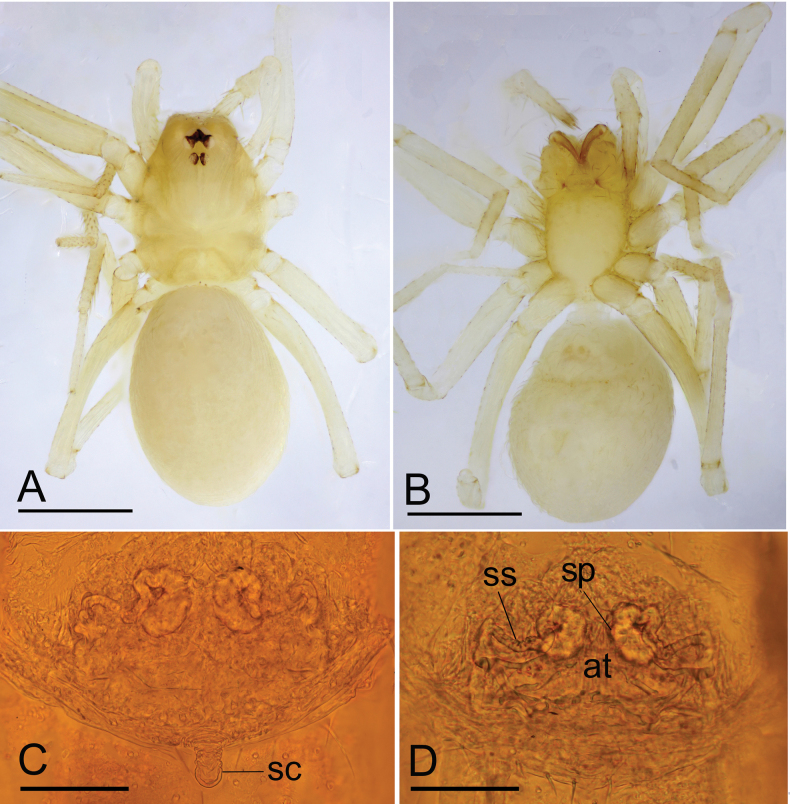
*Jingnetaqishan* sp. nov., female **A, B** habitus, dorsal and ventral views **C, D** genitalia, ventral and dorsal views. Abbreviations: at = atrium; sc = scape; sp.= spermathecae; ss = spermathecal stalk. Scale bars: 0.4 mm (**A, B**); 0.1 mm (**C, D**).

**Figure 4.**
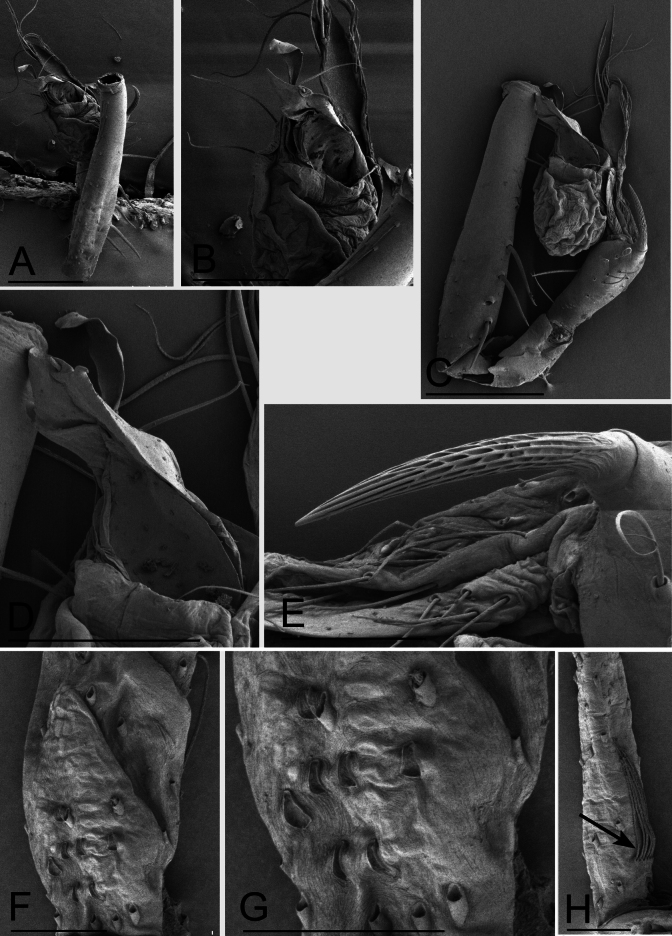
*Jingnetaqishan* sp. nov., SEM **A, C** left palp, ventral and retrolateral views **B** bulb, ventral view **D** distal part of bulb, ventral view **E** detail of palpal tibial apophysis, lateral view **F** patella III, dorsal view **G** detail of patella III, dorsal view **H** metatarsus III, ventral view, arrow shows preening comb. Scale bars: 0.2 mm (**A, C**); 0.1 mm (**B, D**); 0.05 mm (**E–H**).

Figs 1 , 2 , 3 , 4 , 13A , 14

##### Type material.

***Holotype*** China • ♂ (SYNU-1168); Anhui, Chizhou City, Guichi District, Qishan Scenic Area; 30°38'19"N, 117°29'57"E, 70 m; 12.I.2022; H. Fu & K. Yang leg. ***Paratype***: China • ♀ (SYNU-1169), same data as holotype.

##### Etymology.

The specific name refers to the type locality and is a noun in apposition.

##### Diagnosis.

This new species is similar to *Jingnetaexilocula* (Tong and Li 2008: fig. 2A−H) in the horn-shaped apophysis of palpal tibia, but can be distinguished by the chelicerae lacking a stridulatory file on the lateral margin (Fig. 13A) vs. with a stridulatory file, palpal femur with six long setae retrolaterally (Fig. 1D) vs. eight long setae, tip of bulb with a spine-like prolateral sclerite (Fig. 2A, C) vs. lacking and female genital area with a scape (Fig. 3C) vs. lacking.

##### Description.

**Male** (holotype). Habitus as in Fig. 1A, B. Total length 1.55. Carapace 0.62 long, 0.59 wide. Abdomen 0.99 long, 0.68 wide. Eye sizes and interdistances: ALE 0.05, PLE 0.05, PME 0.04; ALE–PME 0.06, PLE–PLE 0.02, PLE–PME 0.03; AER 0.08, PER 0.10. Carapace light yellow. Median groove, cervical grooves and radial furrows distinct. Chelicerae with eight large promarginal and five small retromarginal teeth. Labium rectangular; endites with serrula anterolaterally; sternum light yellow, longer than wide, heart shaped, smooth. Abdomen whitish, ovoid. Leg measurements: I - (1.15, 0.19, 1.16, 0.90, -); II - (0.94, 0.19, 0.95, 0.73, 0.59); III - (-, 0.19, 0.72, 0.64, 0.48); IV - (1.26, -, -, -, -). Metatarsus III with row of ﬁne hairs ventrally (preening comb, arrow in Fig. 4H). Palp (Figs 1C–D, 2A–D, 4A–E): femur with six long setae retrolaterally; tibia with one horn-shaped apophysis distally; tip of bulb with triangular embolus, a spine-like prolateral sclerite and several outgrowths, including a leaf-shaped prolateral outgrowth, a median triangular outgrowth, a small outgrowth and a ribbon-shaped retrolateral outgrowth.

**Female** (paratype). Similar to male in general features. Habitus as in Fig. 3A, B. Total length 1.37. Carapace 0.61 long, 0.49 wide. Abdomen 0.77 long, 0.55 wide. Eye sizes and interdistances: ALE 0.05, PLE 0.05, PME 0.04; ALE–PME 0.10, PLE–PLE 0.03, PLE–PME 0.02; AER 0.08, PER 0.11. Leg measurements: I 2.19 (0.59, 0.21, 0.57, 0.45, 0.37); II 1.85 (0.50, 0.18, 0.46, 0.37, 0.34); III 1.64 (0.45, 0.18, 0.36, 0.35, 0.30); IV 2.38 (0.70, 0.18, 0.69, 0.46, 0.35). Genital area (Fig. 3C) with a scape on the posterior edge. Internal genitalia (Fig. 3D) with a pair of coiled spermathecae and sperm stalk; atrium oval.

##### Distribution.

China (Anhui).

#### ﻿ Jingneta wukuishan

Taxon classificationAnimaliaAraneaeLeptonetidae

Tong sp. nov.

1E044F29-D895-5102-9459-15EDEA2571DC

https://zoobank.org/91782FAE-C3E8-4350-A294-5F2A0DAF7E2F

**Figure 5.**
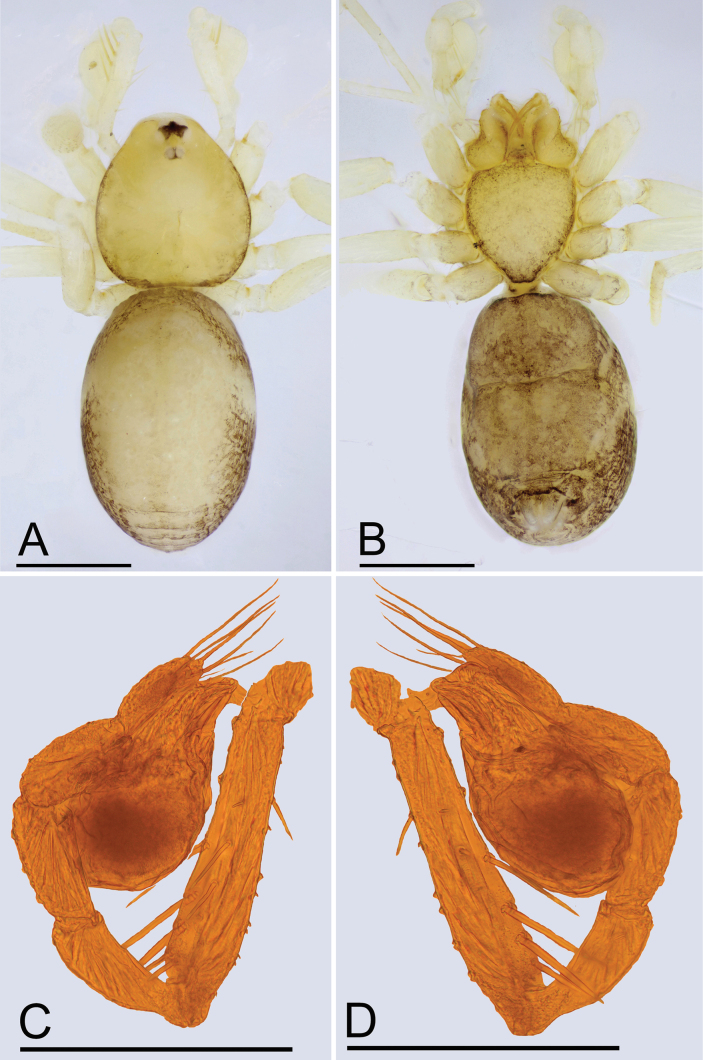
*Jingnetawukuishan* sp. nov., male **A, B** habitus, dorsal and ventral views **C, D** left palp, prolateral and retrolateral views. Scale bars: 0.4 mm (**A, B**); 0.3 mm (**C, D**).

**Figure 6.**
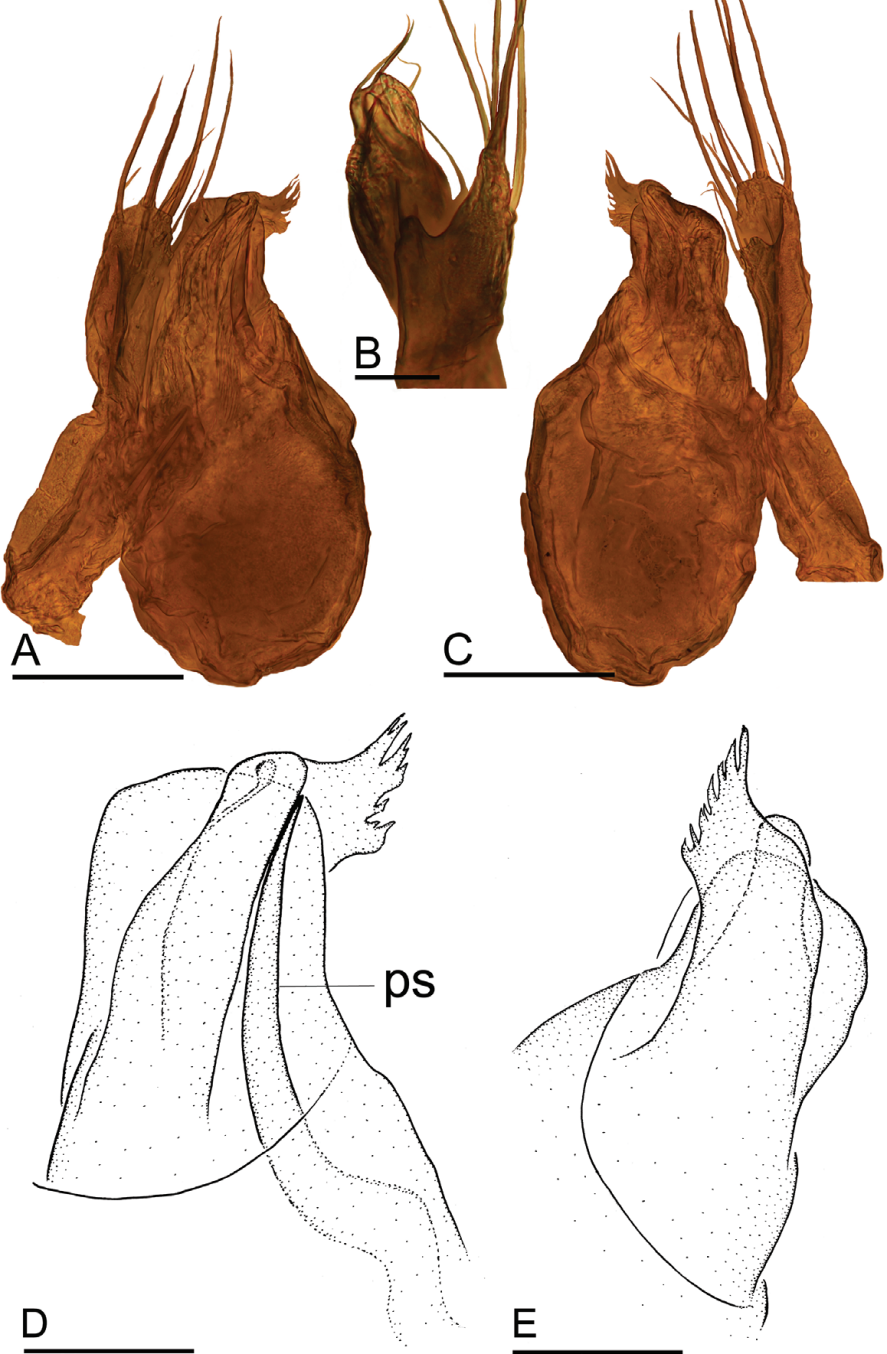
*Jingnetawukuishan* sp. nov. **A, B, C** left palp, prolateral, dorsal and retrolateral views **D, E** distal part of bulb, prolateral and retrolateral views. Abbreviation: ps = prolateral sclerite. Scale bars: 0.1 mm (**A, C**); 0.05 mm (**B, D, E**).

Figs 5 , 6 , 13C , 14

##### Type material.

***Holotype*** China • ♂ (SYNU-1170); Anhui, Huangshan City, She County, Wukui Mountain; 29°51'0"N, 118°24'55"E, 138 m; 3.I.2022; W. Cheng, H. Fu & K. Yang leg. ***Paratype***: China • 1 ♂ (SYNU-1171), same data as holotype.

##### Etymology.

The specific name refers to the type locality and is a noun in apposition.

##### Diagnosis.

This new species is similar to *Jingnetamaculosa* (Song and Xu 1986: fig. 2A−C) in the dark stripes of abdomen, but can be distinguished by the chelicerae with seven promarginal teeth (Fig. 13C) vs. ten promarginal teeth, palpal femur with six long setae retrolaterally and tibia lacking specialized setae (Fig. 5D) vs. nine setae and tibia with three short blunt spines.

##### Description.

**Male** (holotype). Habitus as in Fig. 5A, B. Total length 1.52. Carapace 0.61 long, 0.54 wide. Abdomen 0.93 long, 0.62 wide. Eye sizes and interdistances: ALE 0.06, PLE 0.06, PME 0.05; ALE–PME 0.06, PLE–PLE 0.04, PLE–PME 0.02; AER 0.11, PER 0.12. Carapace yellow to dark brown. Median groove, cervical grooves and radial furrows indistinct. Chelicerae with seven large promarginal and seven small retromarginal teeth. Labium rectangular; endites with serrula anterolaterally; sternum yellow to brown, longer than wide, heart shaped, smooth. Abdomen light brown, darker on sides, ovoid. Leg measurements: I 2.96 (0.83, 0.21, 0.78, 0.65, 0.49); II - (-, -, -, -, -); III 2.73 (0.75, 0.21, 0.67, 0.61, 0.49); IV 3.93 (1.08, 0.21, 1.20, 0.88, 0.56). Palp (Figs 5C, D, 6A–E): femur with six long setae retrolaterally; cymbium constricted medially, attached to a small earlobe-shaped process retrolaterally; tip of bulb with a strong spine-like prolateral sclerite and several membranous outgrowths.

**Female.** Unknown.

##### Distribution.

China (Anhui).

#### ﻿Genus Leptonetela

Taxon classificationAnimaliaAraneaeLeptonetidae

Kratochvíl, 1978

0AE5B04E-F896-54DB-9876-ACEC33316A69

Guineta Lin & Li, 2010: 6.
Qianleptoneta Chen & Zhu, 2008: 12.
Sinoneta Lin & Li, 2010: 82.

##### Type species.

*Sulciakanellisi* Deeleman-Reinhold, 1971.

##### Diagnosis.

See Wang et al. (2017).

##### Composition.

One hundred and twenty-three species, of which 9 species occur in Greece, 2 in Turkey, 1 in Azerbaijan and Georgia, 1 in Vietnam, and 110 species in China, including the one described here.

##### Distribution.

Azerbaijan, China, Georgia, Greece, Turkey and Vietnam.

#### ﻿ Leptonetela jingde

Taxon classificationAnimaliaAraneaeLeptonetidae

Tong sp. nov.

C6F28152-C6FF-5622-B208-9A29BB0BCAF4

https://zoobank.org/E77EDBB0-8D15-4419-8036-F727BE934CC4

**Figure 7.**
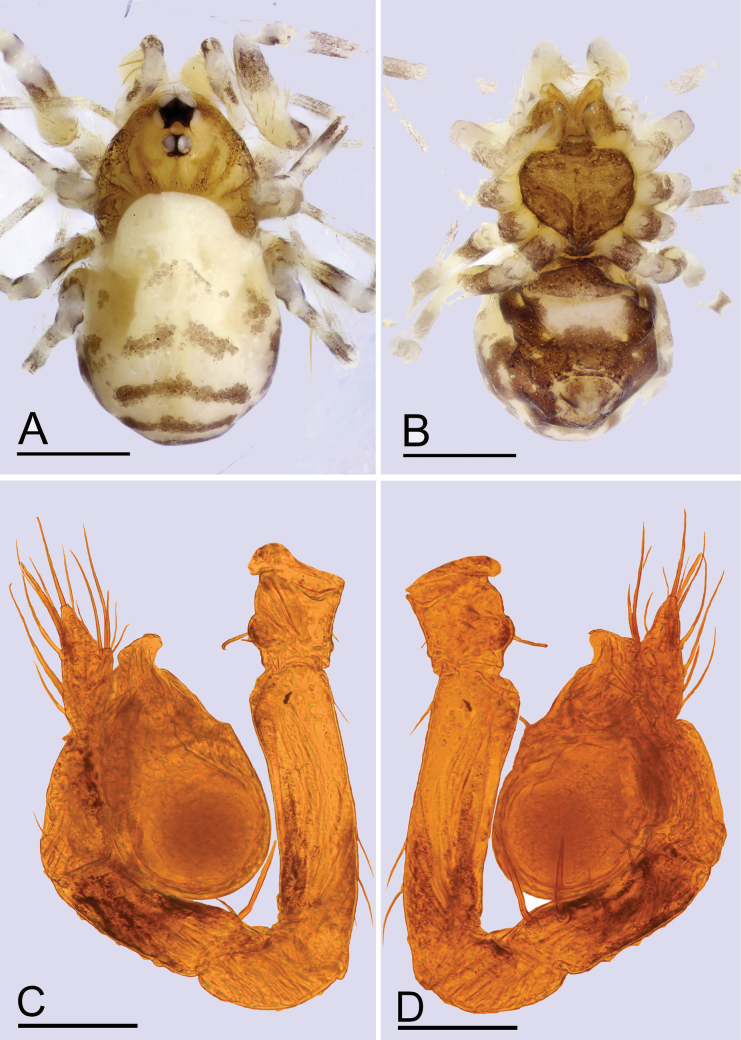
*Leptonetelajingde* sp. nov., male **A, B** habitus, dorsal and ventral views **C, D** left palp, prolateral and retrolateral views. Scale bars: 0.4 mm (**A, B**); 0.1 mm (**C, D**).

**Figure 8.**
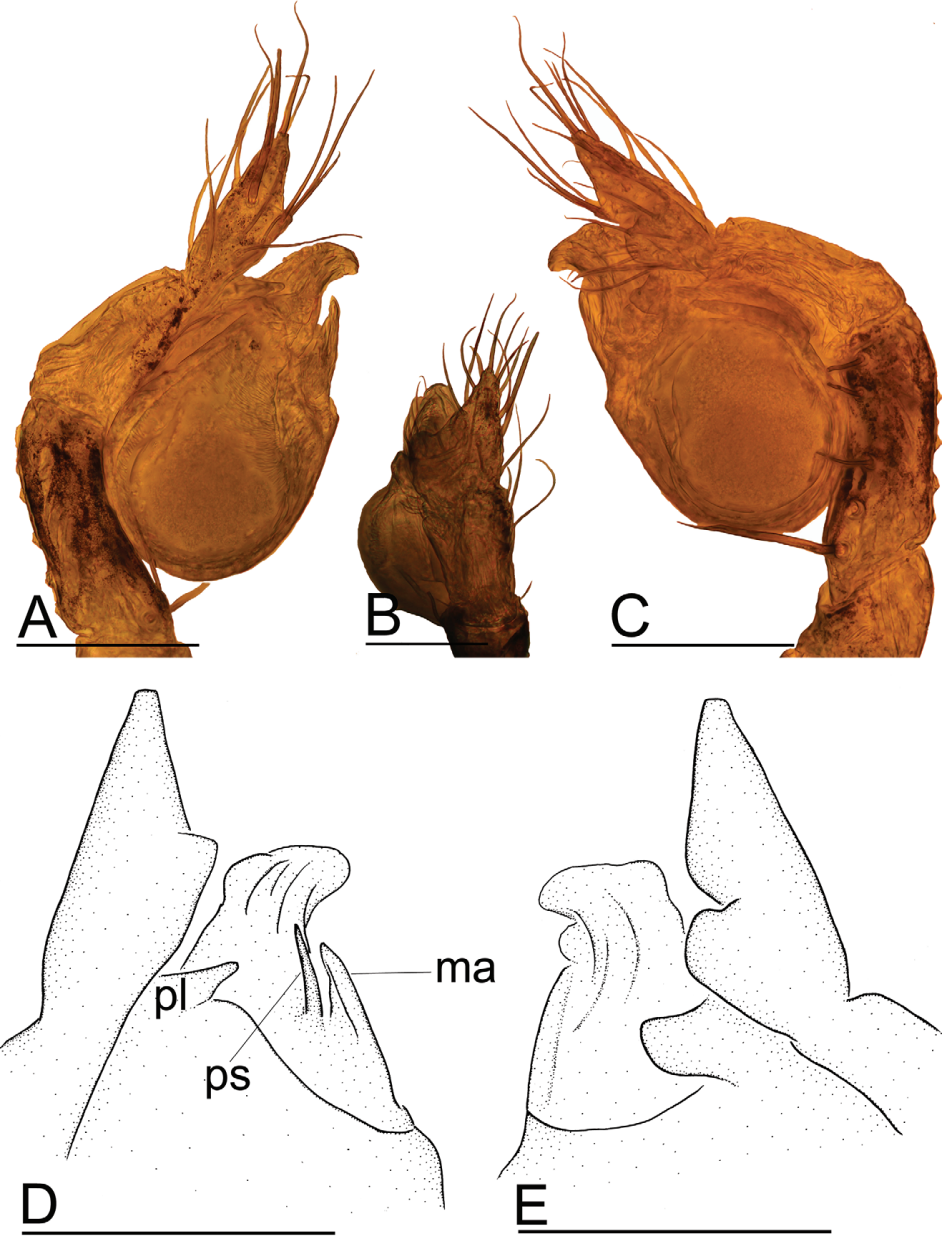
*Leptonetelajingde* sp. nov. **A, B, C** left palp, prolateral, dorsal and retrolateral views **D, E** detail of palpal bulb, prolateral and retrolateral views. Abbreviation: ma = median apophysis; pl = prolateral lobe; ps = prolateral sclerite. Scale bars: 0.1 mm.

**Figure 9.**
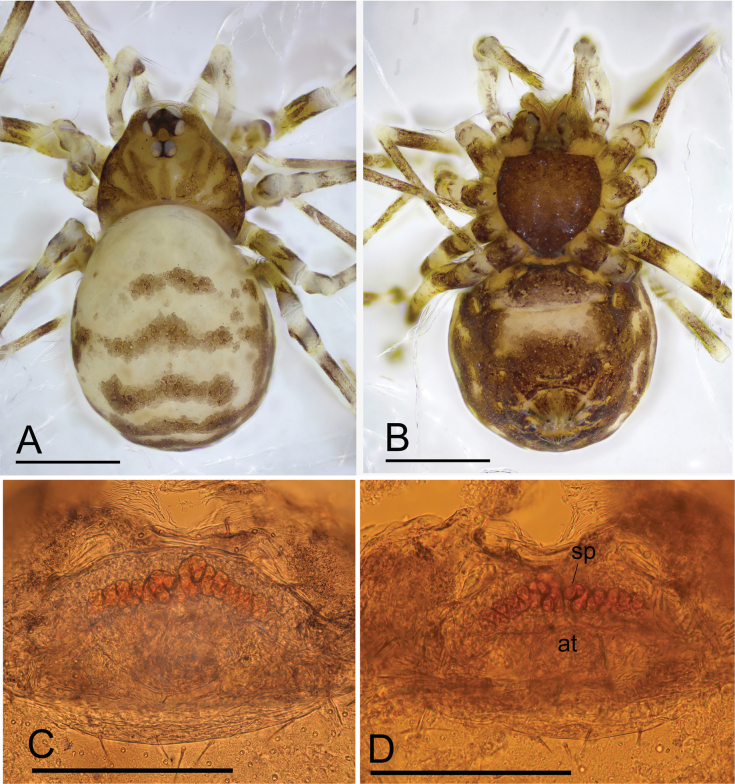
*Leptonetelajingde* sp. nov., female **A, B** habitus, dorsal and ventral views **C, D** genitalia, ventral and dorsal views. Abbreviations: at = atrium; sp.= spermathecae. Scale bars: 0.4 mm (**A, B**); 0.2 mm (**C, D**).

Figs 7 , 8 , 9 , 13B , 14

##### Type material.

***Holotype*** China • ♂ (SYNU-1172); Anhui, Xuancheng City, Jingde County, Tu’er Mountain; 30°18'23"N, 118°32'15"E, 240 m; 7.I.2022; W. Cheng, H. Fu & K. Yang leg. ***Paratype***: China • 4 ♂ 1 ♀ (SYNU-1173–1177), same data as holotype.

##### Etymology.

The specific name refers to the type locality and is a noun in apposition.

##### Diagnosis.

This new species is similar to *Leptonetelamicrodonta* (Wang and Li 2011: figs 28–31) in the long setae on palpal tibia, but can be distinguished by the chelicerae with seven promarginal teeth (Fig. 13B) vs. eight, palpal tibia with four long setae retrolaterally, the basal two thick (Fig. 7D) vs. six long setae, with the basal one thinner and the distal three thick, the prolateral sclerite spine-like (Fig. 8D) vs. fork-shaped, with five teeth distally and the abdomen with four dark chevron-shaped stripes (Figs 7A, 9A) vs. lacking.

##### Description.

**Male** (holotype). Habitus as in Fig. 7A, B. Total length 1.25. Carapace 0.61 long, 0.59 wide. Abdomen 0.89 long, 0.73 wide. Eye sizes and interdistances: ALE 0.08, PLE 0.08, PME 0.07; ALE–PME 0.08, PLE–PLE 0.08, PLE–PME 0.03; AER 0.14, PER 0.17. Carapace light yellow. Median groove, cervical grooves and radial furrows distinct. Chelicerae with seven large promarginal and four small retromarginal teeth, with stridulatory file on the lateral margin. Labium rectangular; endites with serrula anterolaterally; sternum brown, shield shaped, smooth. Abdomen whitish, ovoid, with four dark chevron-shaped stripes. Leg measurements: I 2.66 (0.72, 0.17, 0.73, 0.57, 0.47); II 2.20 (0.62, 0.17, 0.55, 0.46, 0.40); III 1.97 (0.58, 0.18, 0.46, 0.46, 0.29); IV 2.45 (0.72, 0.17, 0.62, 0.54, 0.40). Palp (Figs 7C, D, 8A–E): femur without long setae retrolaterally; tibia with four long setae retrolaterally, the basal two thick; cymbium constricted medially, attached to a large earlobe-shaped process retrolaterally; tip of bulb with a short spine-like prolateral sclerite and leaf-shaped median apophysis.

**Female** (paratype). Similar to male in general features. Habitus as in Fig. 9A, B. Total length 1.36. Carapace 0.64 long, 0.58 wide. Abdomen 0.96 long, 0.80 wide. Eye sizes and interdistances: ALE 0.08, PLE 0.07, PME 0.07; ALE–PME 0.12, PLE–PLE 0.08, PLE–PME 0.02; AER 0.14, PER 0.17. Leg measurements: I - (0.73, 0.20, 0.67, 0.56, -); II 2.19 (0.62, 0.20, 0.52, 0.45, 0.40); III 1.97 (0.58, 0.18, 0.44, 0.45, 0.32); IV - (0.73, 0.21, 0.62, -, -). Internal genitalia (Fig. 9C, D) with sub-trapezoidal atrium, slightly swollen spermathecae, and convoluted spermathecal stalk including six coils.

##### Distribution.

China (Anhui).

#### ﻿Genus Rhyssoleptoneta

Taxon classificationAnimaliaAraneaeLeptonetidae

Tong & Li, 2007

E533DAE5-8571-5E10-8A7C-06E07BACAE7D

##### Type species.

*Rhyssoleptonetalatitarsa* Tong & Li, 2007.

##### Diagnosis.

See Tong and Li (2007) and Wang et al. (2012).

##### Composition.

Three species, including one described here.

##### Distribution.

China (Anhui, Beijing, Hebei).

#### ﻿ Rhyssoleptoneta lishan

Taxon classificationAnimaliaAraneaeLeptonetidae

Tong sp. nov.

7A4AB389-319F-5DDB-A328-AB871236266D

https://zoobank.org/DF212BC3-9B09-4A97-B11F-73669221EAF3

**Figure 10.**
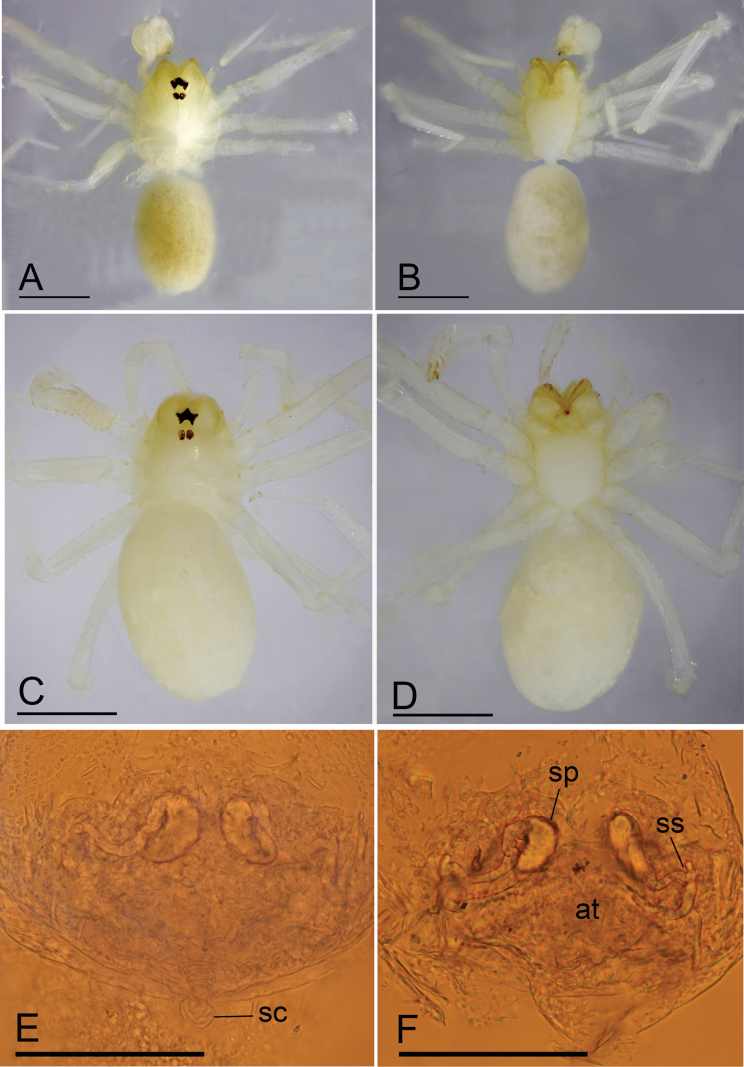
*Rhyssoleptonetalishan* sp. nov. **A, B** male habitus, dorsal and ventral views **C, D** female habitus, dorsal and ventral views **E, F** genitalia, ventral and dorsal views. Abbreviations: at = atrium; sc = scape; sp.= spermathecae; ss = spermathecal stalk. Scale bars: 0.4 mm (**A–D**), 0.1 mm (**E, F**).

**Figure 11.**
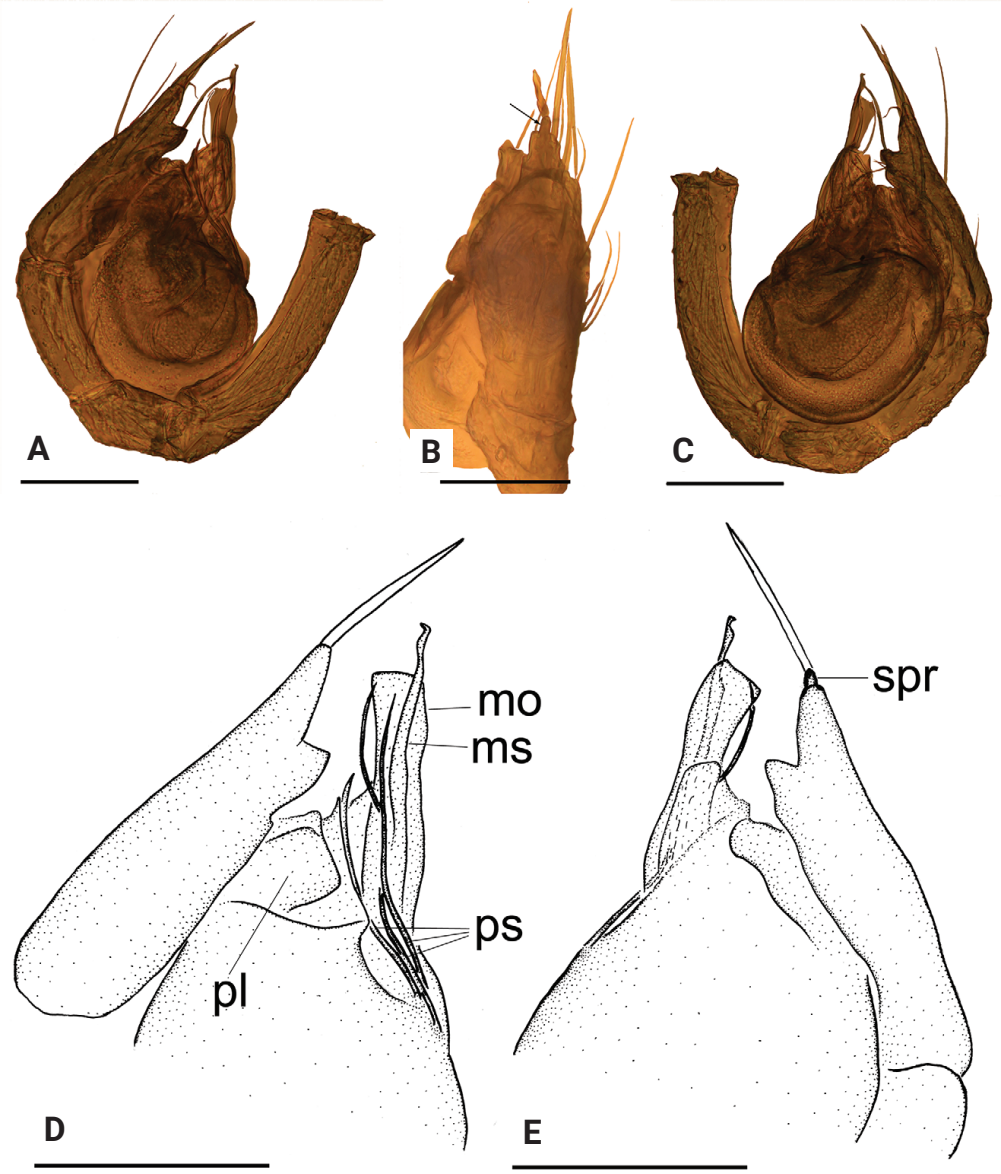
*Rhyssoleptonetalishan* sp. nov. **A, B, C** left palp, prolateral, dorsal and retrolateral views, arrow shows the short projection **D, E** detail of palpal bulb, prolateral and retrolateral views. Abbreviations: mo = median outgrowth; ms = median sclerite; pl = prolateral lobe; ps = prolateral sclerite; spr = short projection. Scale bar: 0.1 mm.

**Figure 12.**
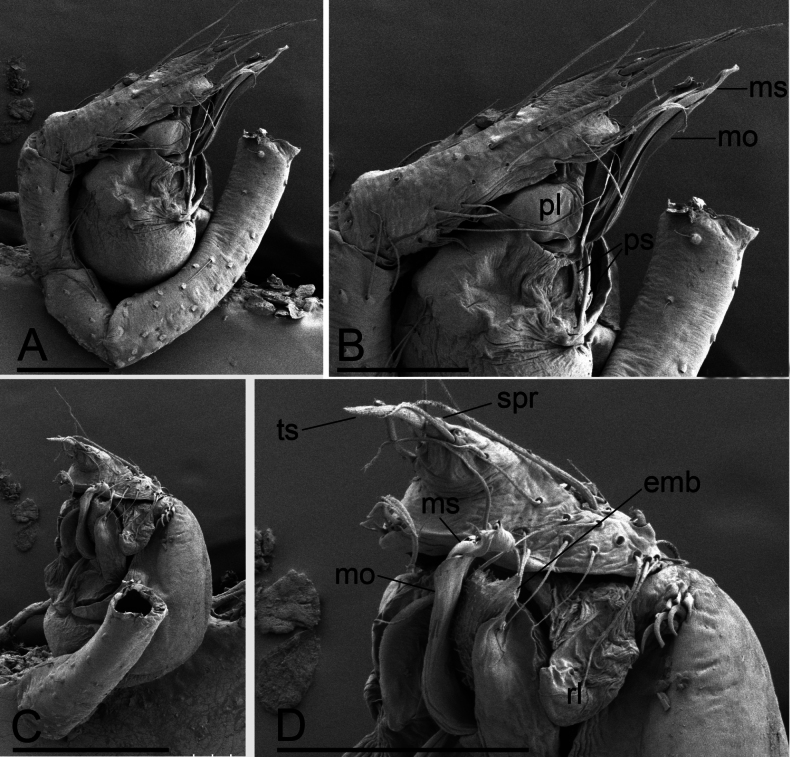
*Rhyssoleptonetalishan* sp. nov., SEM **A, C** left palp, prolateral and dorsal views **B, D** detail of palpal bulb, prolateral and ventral views. Abbreviations: emb = embolus; mo = median outgrowth; ms = median sclerite; pl = prolateral lobe; ps = prolateral sclerite; rl = retrolateral lobe; spr = short projection; ts = tarsal spur. Scale bars: 0.1 mm.

**Figure 13.**
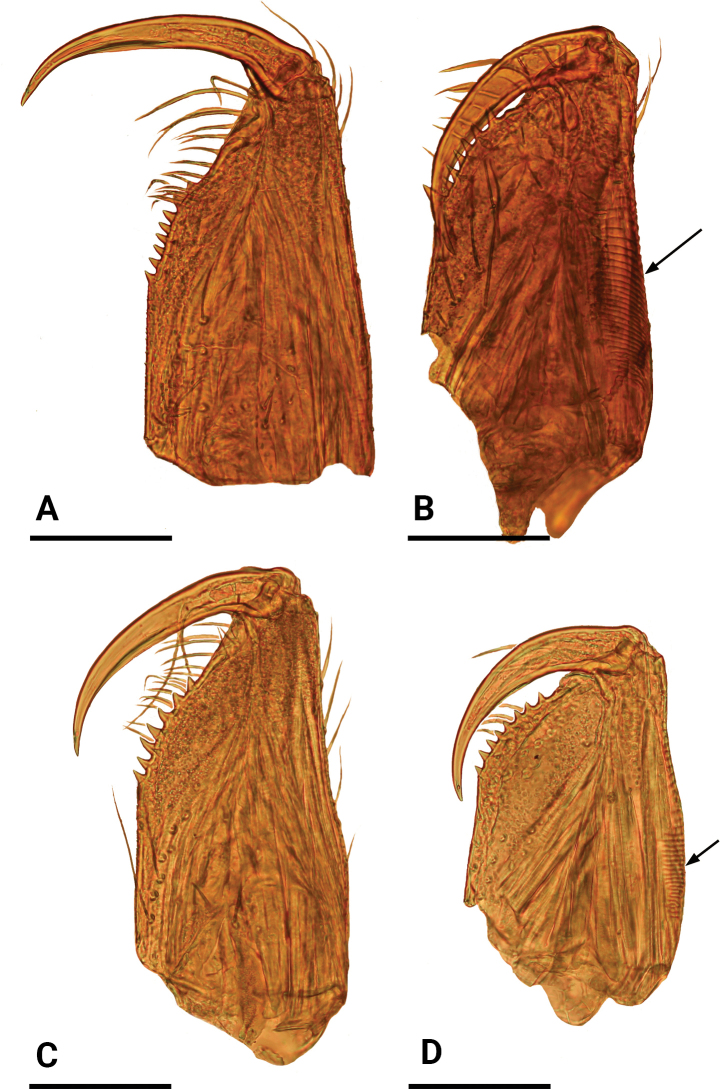
Male left chelicerae, posterior view **A***Jingnetaqishan* sp. nov. **B***Leptonetelajingde* sp. nov. **C***Jingnetawukuishan* sp. nov. **D***Rhyssoleptonetalishan* sp. nov. Arrows show the stridulatory file in B, D. Scale bars: 0.1 mm.

**Figure 14.**
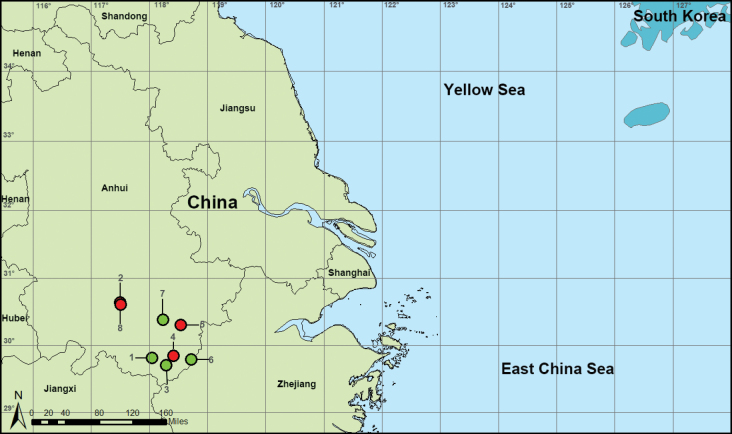
Distribution records of leptonetid spiders from Anhui, China, red circles refer to four new species, green circles indicate four known species **1***Jingnetamaculosa***2***Jingnetaqishan* sp. nov. **3***Jingnetatunxiensis***4***Jingnetawukuishan* sp. nov. **5***Leptonetelajingde* sp. nov. **6***Leptonetelamicrodonta***7***Longileptonetashenxian***8***Rhyssoleptonetalishan* sp. nov.

Figs 10 , 11 , 12 , 13D , 14

##### Type material.

***Holotype*** China • ♂ (SYNU-1163); Anhui, Chizhou City, Guichi District, Lishan Village; 30°36'28"N, 117°30'12"E, 20 m; 14.I.2022; H. Fu & K. Yang leg. ***Paratype***: China • 1 ♀ (SYNU-1164), same data as holotype.

##### Other material examined.

China • 3 ♀; Anhui, Chizhou City, Guichi District, Santaishan Park; 30°39'34"N, 117°28'21"E, 30 m; 13.I.2022; H. Fu & K. Yang leg.

##### Etymology.

The specific name refers to the type locality and is a noun in apposition.

##### Diagnosis.

This new species is similar to *Rhyssoleptonetaaosen* (Zhu and Li 2021: figs 9A–D, 10A–C) in the scape of female genital area, but can be distinguished by the chelicerae with eight promarginal teeth and by the stridulatory file on the lateral margin (Fig. 13D) vs. seven promarginal teeth and lacking the stridulatory file, palpal bulb with membranous median outgrowth, without tooth-shaped projections (Fig. 11D, E) vs. without median outgrowth but with three tooth-shaped projections, and the short projection of the palpal tarsus distally (Fig. 11E) vs. on the middle area.

##### Description.

**Male** (holotype). Habitus as in Fig. 10A, B. Total length 1.31. Carapace 0.62 long, 0.50 wide. Abdomen 0.67 long, 0.45 wide. Eye sizes and interdistances: ALE 0.06, PLE 0.06, PME 0.04; ALE–PME 0.06, PLE–PLE 0.06, PLE–PME 0.02; AER 0.10, PER 0.12. Carapace light yellow. Median groove, cervical grooves and radial furrows indistinct. Chelicerae with eight large promarginal and four small retromarginal teeth, with stridulatory file on the lateral margin. Labium rectangular; endites with serrula anterolaterally; sternum whitish, longer than wide, heart shaped, smooth. Abdomen whitish, ovoid, with four dark chevron-shaped stripes. Leg measurements: I 2.51 (0.70, 0.19, 0.68, 0.55, 0.39); II 2.11 (0.59, 0.19, 0.53, 0.45, 0.35); III 1.80 (0.51, 0.17, 0.45, 0.40, 0.27); IV - (-, -, -, -, -). Male palp (Figs 11A–E, 12A–D): femur without long spines; tibia without special projection; tarsus wide, not branched distally, with a short projection and a long spur distally; bulb complex, wrinkled on prolateral surface, with 3 spine-like prolateral sclerites, with a membranous median outgrowth and belt-like median sclerite; embolus triangular.

**Female** (paratype). Similar to male in general features. Habitus as in Fig. 10C, D. Total length 1.23. Carapace 0.54 long, 0.45 wide. Abdomen 0.80 long, 0.52 wide. Eye sizes and interdistances: ALE 0.05, PLE 0.05, PME 0.04; ALE–PME 0.06, PLE–PLE 0.05, PLE–PME 0.02; AER 0.09, PER 0.11. Leg measurements: I 1.95 (0.55, 0.15, 0.54, 0.38, 0.33); II 1.63 (0.43, 0.15, 0.44, 0.32, 0.29); III 1.44 (0.39, 0.15, 0.32, 0.31, 0.27); IV 2.11 (0.62, 0.16, 0.59, 0.43, 0.31). Genital area (Fig. 10E) with a scape on the posterior edge. Internal genitalia (Fig. 10F) with a pair of coiled spermathecae and sperm stalk; atrium triangular.

##### Distribution.

China (Anhui).

### ﻿Key to leptonetid spiders from Anhui Province, China

Females of *Jingnetawukuishan* are unknown.

**Table:** 

1	Males	**2**
–	Females	**9**
2	Palpal femur with strong spines (Figs 1D, 5D)	**3**
–	Palpal femur without strong spines (Figs 7D, 11A)	**7**
3	Eyes absent; palpal tibia with columnar apophyses; cymbium curved prolaterally, with prolateral spine (Wang et al. 2020: fig. 12D)	***Longileptonetashenxian* Wang & Li, 2020**
–	Eyes present; palpal tibia with one or two horn-shaped or spine-like apophyses; cymbium branched distally, without prolateral spine (Figs 1D, 2B)	**4**
4	Body light yellow, abdomen without dark stripes (Fig. 1A)	**5**
–	Body dark brown, abdomen with dark stripes (Fig. 5A)	**6**
5	Male palpal tibia with one horn-shaped apophysis distally (Fig. 2B)	***Jingnetaqishan* sp. nov.**
–	Male palpal tibia without horn-shaped apophysis (Song and Xu 1986: fig. 1C)	***Jingnetatunxiensis* (Song & Xu, 1986)**
6	Chelicerae with ten promarginal teeth; palpal femur with nine long setae retrolaterally and tibia with three short blunt spines (Song and Xu 1986: fig. 2C)	***Jingnetamaculosa* (Song & Xu, 1986)**
–	Chelicerae with seven promarginal teeth (Fig. 13C); palpal femur with six long setae retrolaterally and tibia lacking specialized setae (Fig. 5D)	***Jingnetawukuishan* sp. nov.**
7	Palpal tibia without strong spines (Fig. 11A)	***Rhyssoleptonetalishan* sp. nov.**
–	Palpal tibia with a row of strong spines (Fig. 7D)	**8**
8	Abdomen with dark chevron-shaped stripes (Fig. 7A); chelicerae with seven promarginal teeth (Fig. 13B); palpal tibia with four long setae retrolaterally, the basal two thick (Fig. 7D); prolateral sclerite spine-like (Fig. 8D)	***Leptonetelajingde* sp. nov.**
–	Abdomen without dark chevron-shaped stripes; chelicerae with eight promarginal teeth (Wang and Li 2011: figs 28A, 31C); palpal tibia with six long setae retrolaterally, the basal one thinner and the distal three thick (Wang and Li 2011: figs 28D, 30B); prolateral sclerite fork-shaped, with five teeth distally (Wang and Li 2011: figs 28B, 31D)	***Leptonetelamicrodonta* (Xu & Song, 1983)**
9	Abdomen with dark chevron-shaped stripes (Fig. 9A)	**10**
–	Abdomen without dark chevron-shaped stripes	**11**
10	Abdomen with four dark chevron-shaped stripes; spermathecal stalk including six coils (Fig. 9D)	***Leptonetelajingde* sp. nov.**
–	Abdomen with three dark chevron-shaped stripes; spermathecal stalk straight, without coils (Song and Xu 1986: fig. 2B)	***Jingnetamaculosa* (Song & Xu, 1986)**
11	Eyes absent (Wang et al. 2020: fig. 13A)	***Longileptonetashenxian* Wang & Li, 2020**
–	Eyes present	**12**
12	Genital area with a scape (Fig. 3C)	**13**
–	Genital area without a scape	**14**
13	Scape enlarged distally; atrium triangular (Fig. 10F)	***Rhyssoleptonetalishan* sp. nov.**
–	Scape not enlarged distally; atrium oval (Fig. 3D)	***Jingnetaqishan* sp. nov.**
14	Carapace with brown lateral margin; spermathecal stalk including six coils (Wang and Li 2011: figs 29C, 30B)	***Leptonetelamicrodonta* (Xu & Song, 1983)**
–	Carapace without brown lateral margin; spermathecal stalk straight, without coils (Song and Xu 1986: fig. 1B)	***Jingnetatunxiensis* (Song & Xu, 1986)**

## Supplementary Material

XML Treatment for Jingneta

XML Treatment for Jingneta qishan

XML Treatment for Jingneta wukuishan

XML Treatment for Leptonetela

XML Treatment for Leptonetela jingde

XML Treatment for Rhyssoleptoneta

XML Treatment for Rhyssoleptoneta lishan

## ﻿Additional information

### Author ORCIDs

Shuhui Li https://orcid.org/0009-0000-0266-6859

Qiang Chen https://orcid.org/0009-0007-9722-1561

Yanfeng Tong https://orcid.org/0000-0002-4348-7029

### Data availability

All of the data that support the findings of this study are available in the main text.
